# Effects of Manipulating Fibroblast Growth Factor Expression on Sindbis Virus Replication In Vitro and in *Aedes aegypti* Mosquitoes

**DOI:** 10.3390/v12090943

**Published:** 2020-08-26

**Authors:** Wenbi Wu, Cody A. Simmons, Jessica Moffitt, Rollie J. Clem, A. Lorena Passarelli

**Affiliations:** 1 School of Life Sciences, Sun Yat-sen University, Guangzhou 510275, China; wuwenbi3@mail.sysu.edu.cn; 2 Division of Biology, Kansas State University, Manhattan, KS 66506, USA; cody.simmons@bms.com (C.A.S.); jessicadmoffitt@gmail.com (J.M.); 3 Cody A. Simmons, Bristol Myers Squibb, Devens, MA 01434, USA

**Keywords:** arbovirus, alphavirus, transmission, midgut escape barrier, basal lamina, vector biology, vector competence

## Abstract

Fibroblast growth factors (FGFs) are conserved among vertebrate and invertebrate animals and function in cell proliferation, cell differentiation, tissue repair, and embryonic development. A viral fibroblast growth factor (vFGF) homolog encoded by baculoviruses, a group of insect viruses, is involved in escape of baculoviruses from the insect midgut by stimulating basal lamina remodeling. This led us to investigate whether cellular FGF is involved in the escape of an arbovirus from mosquito midgut. In this study, the effects of manipulating FGF expression on Sindbis virus (SINV) replication and escape from the midgut of the mosquito vector *Aedes aegypti* were examined. RNAi-mediated silencing of either *Ae. aegypti* FGF (AeFGF) or FGF receptor (AeFGFR) expression reduced SINV replication following oral infection of *Ae. aegypti* mosquitoes. However, overexpression of baculovirus vFGF using recombinant SINV constructs had no effect on replication of these viruses in cultured mosquito or vertebrate cells, or in orally infected *Ae*. *aegypti* mosquitoes. We conclude that reducing FGF signaling decreases the ability of SINV to replicate in mosquitoes, but that overexpression of vFGF has no effect, possibly because endogenous FGF levels are already sufficient for optimal virus replication. These results support the hypothesis that FGF signaling, possibly by inducing remodeling of midgut basal lamina, is involved in arbovirus midgut escape following virus acquisition from a blood meal.

## 1. Introduction

Fibroblast growth factor (FGF) homologs are ubiquitous in metazoans and the FGF signaling pathway is involved in a variety of processes such as angiogenesis, wound healing, and embryonic development [1]. When released from cells, FGF binds to the FGF receptor (FGFR) and initiates signaling through Ras-MAPK and other pathways. While mammals encode numerous homologs of FGF and FGFR, most insects, such as *Drosophila melanogaster*, contain only one copy of each gene. In *Drosophila*, FGF and FGFR have been shown to play important roles in the development of the tracheal system during embryonic development and later in developing larvae [2]. 

One process that is known to be triggered by FGF signaling is remodeling of basal lamina [3], part of the extracellular matrix that is secreted by epithelial cells and is mainly composed of type IV collagen, laminin, nidogen, and perlecan proteoglycans. Basal laminae provide support for epithelia and can serve as barriers to prevent the passage of pathogens. In insects, the tracheal network, consisting of epithelial-lined chitinous tubes, provides gas exchange and tracheae that are closely associated with all organs including the midgut. In order for tracheal cells to migrate or extend towards hypoxic tissues, they must first degrade their basal lamina. Once migration is complete, the basal lamina is resynthesized. 

The *Baculoviridae*, a family of large DNA viruses that infect mainly lepidopteran insects, is the only group of viruses known to encode homologs of FGF. Baculovirus-encoded FGF homologs, called viral fibroblast growth factor (vFGFs), are not essential for baculovirus replication per se, but expression of vFGF enhances baculovirus systemic infection by inducing the dismantling of basal lamina of tracheal epithelial cells closely associated with the midgut. This allows baculovirus to escape from the midgut by infecting tracheal epithelial cells, leading to efficient systemic infection in other organs [3]. 

The mechanism by which vFGF enhances midgut escape was determined by a series of studies that showed: (1) baculovirus-encoded vFGFs have properties similar to those of cellular FGFs, including secretion, binding heparin, and inducing chemotaxis [4,5,6]; (2) both partially purified vFGF protein and vFGF that is associated with baculovirus virions stimulate lepidopteran cell motility in vitro [4,5,7]; (3) in two different baculoviruses, deletion of vFGF results in slower infection or mortality of lepidopteran insect hosts than vFGF expressing viruses [8,9,10,11]; and (4) oral infection with baculovirus initiates a cascade of proteinase activation involving vFGF signaling, activation of effector caspases and matrix metalloproteinases, and cleavage of basal lamina proteins, resulting in basal lamina remodeling [3,9]. Mutant baculoviruses lacking vFGF are delayed in causing basal lamina remodeling and in their ability to escape the midgut following oral infection [3]. In contrast, baculovirus infection by intrahaemocoelic injection does not lead to detectable changes in tracheal basal lamina [12]. 

When arboviruses are ingested by female mosquitoes along with a blood meal, they encounter barriers that are similar to those faced by baculoviruses in lepidopteran insects. In certain cases, infection of a mosquito by an arbovirus results in established midgut infection but lack of a disseminated infection, which has led to the concept of a midgut escape barrier [13,14,15]. It is thought that the basal lamina surrounding the midgut prevents passive diffusion of virus particles [16]. This hypothesis has recently gained additional support from studies showing that blood feeding results in thinning or micro-disruptions in the midgut basal lamina [17,18,19]. These structural changes were associated with increased collagen IV degradation, indicative of active basal lamina remodeling. Furthermore, when *Aedes aegypti* mosquitoes that had been orally infected were given a second, non-infectious, blood meal, dissemination of chikungunya virus, Zika virus, and dengue virus was increased [18,19]. Together these recent results suggest that blood feeding is accompanied by active basal lamina remodeling. However, many questions remain about the nature of the midgut escape barrier and the mechanisms of arbovirus midgut escape, since blood feeding does not allow escape of all viruses from the mosquito midgut (for example in cases where a midgut escape barrier naturally exists). 

Sindbis virus (SINV) (*Togaviridae*; *Alphavirus*) is considered the prototype alphavirus and has long been used as a model to study arbovirus-vector interactions. SINV transducing systems have been developed that allow expression of genes of interest in mosquito or vertebrate cells, as well as in mosquitoes or vertebrate hosts [20,21,22,23,24,25,26,27]. One type of SINV transducing system utilizes a duplicated copy of the viral subgenomic promoter, which normally drives expression of the structural genes, to drive expression of genes of interest. TE5’2J (herein called TE) [24] is a double subgenomic promoter SINV constructed from a chimeric mouse neurovirulent variant of the African strain AR339, named TE12 [28,29]. Another double subgenomic promoter SINV infectious clone, 5’dsMRE16ic (herein called MRE), was constructed from the MRE16 strain of SINV, which was isolated from mosquitoes in Malaysia and minimally passaged in cell culture [30]. While TE5’2J replicates well in cultured mosquito cells, it is less efficient at infecting mosquito midguts and disseminating from the midgut than 5’dsMRE16ic after oral infection [30,31]. 

Following virus uptake in a blood meal, SINV initially infects the mosquito foregut and midgut. To establish a systemic infection, the virus must escape the midgut. Since baculovirus vFGF enhances baculovirus midgut escape, we sought to investigate whether FGF signaling plays a role in SINV oral infection of mosquitoes by SINV. 

## 2. Methods

### 2.1. Cell Culture

The Sf9 insect cell line, a clonal isolate of IPLB-Sf21-AE cells, derived from the fall armyworm *Spodoptera frugiperda* [32], was maintained in TC-100 medium (Invitrogen, Carlsbad, CA, USA) supplemented with 10% fetal bovine serum (FBS), penicillin G (60 µg/mL), streptomycin sulfate (200 µg/mL), and amphotericin B (0.5 µg/mL). Baby hamster kidney-derived BHK-21 cells were maintained in Dulbecco’s modified Eagle’s medium (DMEM) (Gibco/Thermo Fisher Scientific, Waltham, MA, USA) supplemented with 10% FBS. *Ae. albopictus*-derived C6/36 cells were maintained in Leibovitz’s medium (Gibco/Thermo Fisher Scientific) containing 10% FBS. *Ae. aegypti*-derived Aag2 cells were maintained in Schneider’s *Drosophila* medium (Gibco/Thermo Fisher Scientific) containing 10% FBS. Sf9 cells, C6/36 cells, and Aag2 cells were cultured at 27 °C, and BHK-21 cells were cultured at 37 °C with 5% CO_2_.

### 2.2. Insects

*Ae. aegypti* mosquitoes, strain ‘Orlando’, (obtained from James Becnel, USDA, Agricultural Research Service in Gainesville, FL, USA) were reared in an incubator at 27 °C with 80% humidity and under a 12-h light/12-h dark cycle. They were fed ad libitum on raisins and water prior to and post-blood meals. 

### 2.3. Quantitative RT-PCR

Total RNA was isolated from homogenized midgut tissues of approximately 10 pooled individual mosquitoes or from Aag2 cells using Trizol reagent (Invitrogen) and treated with RNase-free DNase. Total RNA (2 µg) was used to synthesize cDNA using an oligo-d(T) primer. The resulting cDNA was analyzed by qPCR using primers specific for *Ae. aegypti* FGF (F-GACGCACGACGATCA, R-GAATGCTGCGTCGAG), FGFR (F-ACCGCCGTTGTCAGCACTAATGTT, R-TCACCCCTGGGATGCTGTTCTCTA), or rpS7 (F-GCGTGGAGCGATTGATTTC, R-CCGCATGTTGTCTTTACTGTCTTTG). Relative expression values were calculated by the comparative C_T_ method [33], using rpS7 as the internal control.

### 2.4. Transient Gene Silencing in Aag2 Cells and Mosquitoes

PCR was used to amplify a 471 bp fragment of the Aefgf ORF using primers (F-AAAACGCCGTCAATCAATCAGTA, R-CAGCGGCCACAGTCGTTC), containing the T7 promoter at the 5′ ends. Similarly, a 451 bp fragment of the Aefgfr ORF was amplified using primers (F-CCCGCGTGGCTAATCTG, R-TCGTCCGGTTCTTCATCGTC). The resulting amplified DNAs were used to synthesize dsRNA using an Ampliscribe T7 high-yield transcription kit (Epicentre Biotechnologies, Madison, WI, USA) according to the protocol of the manufacturer. The dsRNAs were purified and added to Aag2 cells or intrathoracically injected into mosquitoes as previously described [26,34].

### 2.5. Purification of vFGF and Transmigration Experiments

Sf9 cells were transfected with the vFGF-expressing plasmid pHSFGFHA [4] or pBlueScript II SK+ (Stratagene/Sigma Aldrich) as a control, using a liposome preparation as previously described [35]. Cells were maintained in the liposome-DNA mix for 5 h at 27 °C. Cells were then washed twice with TC-100 media and maintained in TC-100 media at 27 °C. Twenty hours post-transfection, cells were incubated at 42 °C for 30 min to induce expression of vFGF from the *Drosophila* heat shock promoter (hsp70). Cells were maintained at 27 °C for another 4 h before cells and supernatants were collected. 

vFGF was partially purified from the supernatant as previously described [4]. Briefly, supernatant from transfected cells was incubated with heparin-Sepharose 6 Fast Flow beads (Amersham Biosciences/GE Healthcare, Chicago, IL, USA) and proteins were eluted with phosphate buffer containing 30% glycerol and 1.2 M NaCl. Supernatant from the pBlueScript-transfected cells was treated similarly and used in the transmigration assay.

The cell transmigration experiment was performed as previously described [4] using Costar transwells with polycarbonate membrane inserts. Approximately 1 × 10^5^ Aag2 or C6/36 cells were seeded onto 3 µM pore-size transwell inserts. Partially purified vFGF or control proteins were added to 24-well plates containing phosphate buffered saline. The transwell inserts were transferred to 24-well plates containing heparin-purified proteins and incubated at 27 °C for 4 h before removal. Cells that had migrated into the wells were quantified using CellTiter-Glo luminescent substrate (Promega, Madison, WI, USA), according to the provided protocol. 

### 2.6. Recombinant SINV Construction

The baculovirus AcMNPV *vfgf* gene containing an HA epitope tag at the C terminus was amplified by PCR using pHSFGFHA [4] as a template and inserted in sense and antisense orientations downstream of the duplicated, subgenomic promoter sequences into the infectious cDNA clones 5’dsMRE16ic or TE5’2J of SINV [24,30,31]. 

Two primers, HASTOP R (5′-TTAGGCGTAATCTGGGACGTC-3′) and FGFXbaI5′ (5′-TCTAGAATGTATCGCTTGCTGGCACT-3′), were used to PCR-amplify vFGFHA. The resulting amplicon was ligated into pCR II (Invitrogen) to generate the plasmid pCRII 5′-XbaI AcFGF-HA Stop-3′. Plasmid DNA was sequenced to confirm the correct nucleotide sequence. The vFGFHA insert was released by digestion with *Eco*RI and the ends were blunt-ended with DNA polymerase I, Large (Klenow) Fragment (New England Biolabs, Ipswich, MA, USA), and DNA purified. 

To generate the TE/vFGF recombinant virus, TE5′2J was digested with XbaI, blunt-ended with Klenow Fragment, dephosphorylated, and gel-purified. The purified vFGFHA fragment was then ligated with TE5′2J to generate TE/vFGF or TE/vFGFas, containing the sense or antisense orientations of vFGFHA, respectively. To generate the MRE/vFGF recombinant virus, 5′dsMRE16ic was digested with PmeI, dephosphorylated, and gel-purified. The vFGFHA fragment was ligated with 5’dsMRE16ic to generate MRE/vFGF or MRE/vFGFas with vFGFHA in the sense or antisense orientations, respectively. 

### 2.7. Viral RNA Transfection, Virus Infection, and Viral Growth Curves Analyses

Viral RNA was produced from infectious cDNA clones using the AmpliScribe SP6 High Yield Transcription Kit (Epicentre Biotechnologies) and m^7^G (5′)ppp(5′)G Cap analogue (Ambion/Thermo Fisher Scientific), according to the protocol of the manufacturer. Viral RNA transfections of BHK-21 cells were carried out as previously described [25]. Virus-containing supernatant was collected at 5 dpi, aliquoted, and stored at −80 °C (Passage 1 or P1 virus). P1 virus was amplified once in C6/36 cells to obtain a P2 virus (Passage 2) stock, which was aliquoted and stored at −80 °C. All the viruses used in this study were P2 virus stocks obtained from C6/36 cells and were thawed only once prior to use.

To perform viral growth curves, Aag2, BHK-21, and C6/36 cells were infected with SINV recombinants at an MOI of 0.001 PFU/cell and virus-containing supernatants were collected at the designated time points pi. Virus titers were determined by TCID_50_ end-point dilution assays [36] using BHK-21 cells. 

### 2.8. Plaque Purification

Plaque purification was performed to estimate the proportion of viruses in the P2 virus stock that expressed vFGF during infection. BHK-21 cells were infected with TE/vFGF P2 virus at an MOI of 0.001 PFU/cell in a 100-mm plate. One hour post-absorption, cells were washed with DMEM medium and then overlaid with 15 mL of DMEM medium containing 10% FBS and 0.5% agarose pre-warmed to 42 °C, followed by incubation for 3 days before staining with neutral red solution (Sigma-Aldrich, St. Louis, MO, USA). Virus plaques were picked and amplified once in BHK-21 cells in a 24-well plate. Supernatants of the infected cells were collected at 3 dpi and were subjected to immunoblotting to determine viruses that expressed vFGF.

### 2.9. Immunoblotting

To detect recombinant vFGF expression, Aag2, BHK-21, and C6/36 cells were infected at an MOI of 5 PFU/cell. At designated time points, supernatants and cells were collected and subjected to immunoblotting. To detect vFGF expression in midguts, mosquitoes were dissected, and midguts were collected, washed with PBS, briefly sonicated, and subjected to immunoblotting. Protein samples were mixed with 2× PLB (Protein Loading Buffer: 0.25 M Tris-Cl, pH 6.8, 4% SDS, 20% glycerol, 10% 2-mercaptoethanol and 0.02% bromophenol blue) and incubated at 100 °C for 5 min. Proteins were analyzed by sodium dodecyl sulfate-polyacrylamide gel electrophoresis (SDS-PAGE), transferred onto PVDF membrane (MilliporeSigma, Burlington, MA, USA), and probed with one of the following primary antibodies: (i) mouse monoclonal anti-HA antibody (Covance, Princeton, NJ, USA); (ii) rabbit polyclonal anti-collagen IV antibody (Abcam, Cambridge, MA, USA); (iii) rabbit polyclonal anti-laminin antibody (Abcam); (iv) mouse monoclonal anti-β-actin antibody (Sigma-Aldrich), followed by incubation with horseradish peroxidase-conjugated secondary antibodies (Sigma-Aldrich). Blots were developed using the SuperSignal West Pico Chemiluminescent substrate (Pierce Biotechnology/Thermo Fisher Scientific) and exposed to X-ray films.

### 2.10. Caspase Activity Assays

Aag2 or C6/36 cells (1 × 10^6^) were seeded in 35-mm diameter culture dishes and infected at an MOI of 5 PFU/cell. Cells were collected, washed with PBS and lysed in caspase assay buffer (20 mM HEPES KOH, pH 7.5, 50 mM KCL, 1.5 mM MgCl_2_,1 mM EDTA, 1 mM EGTA, 1mM DTT, 250 mM sucrose) to measure caspase activity using the mammalian caspase-3 substrate N-acetyl-Asp-Glu-Val-Asp-(7-amino-4-trifluoromethylcoumarin) (DEVD-AFC; MP Biomedicals, Santa Ana, CA, USA) as described previously [25]. 

Mosquitoes were dissected and midguts were collected at different time points post blood-feeding with or without virus. Midguts were washed with PBS and disrupted by sonication to measure caspase activity.

### 2.11. Cell Viability Assays

C6/36 cells (1 × 10^5^) were seeded in a 96-well plate and infected at an MOI of 5 PFU/cell. At designated time points, CellTiter-Glo luminescent substrate (Promega) was added to quantify the number of viable cells according to the manual. Luminescence was recorded using a Wallac Victor^3^ 1420 Multilabel counter (PerkinElmer, Waltham, MA, USA).

### 2.12. DNA Fragmentation Assays

C6/36 cells (2 × 10^6^) were seeded in 6-well plates and infected with recombinant SINV at an MOI of 5 PFU/cell. At different times pi, cells were collected, and total cellular DNA was prepared with the Wizard Genomic DNA Purification kit (Promega), according to the protocol of the manufacturer. Aliquots of each sample were separated by agarose gel electrophoresis and stained with ethidium bromide. 

### 2.13. Oral Infection of Mosquitoes

To orally infect mosquitoes, tissue culture supernatant containing virus (5 × 10^6^ PFU/mL for MRE-based viruses or 2 × 10^8^ PFU/mL for TE-based viruses) were mixed 1:1 with defibrinated sheep blood (Colorado Serum Company, Denver, CO, USA) and blood feeding was performed as previously described [26]. Female mosquitoes (maintained with 10% male mosquitoes) were given water only after eclosion, and water was removed 3 to 6 h prior to being given a blood meal two to three days post-eclosion. Following blood feeding, engorged female mosquitoes were collected and maintained with raisins and water until experiments were completed. At designated time points, blood-fed mosquitoes were collected for whole body virus titration, or dissected to separate midgut and carcass for virus titration, caspase activity, or immunoblotting assays. 

### 2.14. Detection of Viral Antigen in Mosquito Midguts 

To detect SINV antigen in midguts, tissues were dissected and fixed in 4% paraformaldehyde (in PBS) at room temperature (RT) for 2 h. The fixed midguts were washed two times in PBT (PBS containing 0.1% Triton X-100) for 5 min per wash at RT, followed by permeabilization with ice-cold methanol and incubation at −20 °C for 15 min. Midguts were then washed with PBT containing 1% DMSO at RT for 15 min before incubation with blocking buffer (10% FBS and 1% BSA in PBS) for 1 to 2 h at RT. Midguts in blocking buffer were then incubated with 1:100 blocking buffer-diluted anti-SINV E1 monoclonal antibody 30.11 (obtained from Stephen Higgs, Kansas State University) overnight at 4 °C. Midguts were then washed three times in PBT for 30 min per wash, followed by incubation with goat anti-mouse Alexa Fluor 488-conjugated secondary antibody (Molecular Probes) diluted 1:500 in blocking buffer and incubated for 1 h in the dark at RT. Samples were kept in the dark for the remaining procedures. Midguts were washed three times in PBT (30 min per wash) before being mounted on glass slides using a coverslip and Fluoromount-G (Southern Biotech) and stored at 4 °C in the dark until visualized by confocal fluorescence microscopy. 

The extent of midgut infection was scored as previously described [26,31], determined by multiplying the estimated percentage of the midgut surface area that was infected by the brightness of the antibody staining using an arbitrary scale of 1 to 3 (1 = dim, 2 = moderate, 3 = bright). Samples were blinded and randomized, and were independently assessed by two individuals, with similar results.

### 2.15. TCID_50_ Assay with SINV-Infected Mosquito Samples

To analyze SINV replication in mosquitoes, female mosquitoes were orally infected as described above. At designated time points, mosquitoes were collected, homogenized by triturating with a micro-pestle in a microcentrifuge tube containing 250 µL DMEM medium supplemented with ampicillin (15 µg/mL), streptomycin sulfate (15 µg/mL), and gentamicin sulfate (10 µg/mL). Homogenized tissues were pelleted, and supernatants were subjected to TCID_50_ assay using BHK-21 cells. Infection was determined by the presence of cytopathic effect. In some cases, mosquitoes were dissected to separate carcass and midguts for TCID_50_ assays as described above. 

## 3. Results

### 3.1. Role of Endogenous AeFGF and AeFGFR in SINV Infection

**Figure 1:**
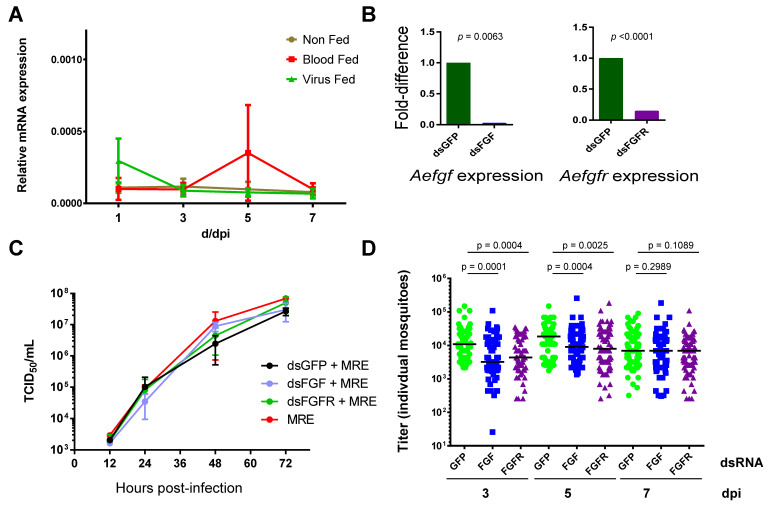
Effect of silencing AeFGF or AeFGFR expression on SINV replication in mosquitoes. (**A**) Ingestion of a blood meal, with or without SINV, does not affect FGF expression in midgut at the mRNA level. Two day-old adult female *Ae. aegypti* were either fed only raisins and water (Non Fed), fed a blood meal mixed 1:1 with cell culture media (Blood Fed), or fed a blood meal mixed 1:1 with cell culture media containing SINV (MRE5′2J strain or MRE; Virus Fed). At 1, 3, 5, and 7 days later, midguts were harvested and RNA from pools of 10 midguts was used in qRT-PCR assays to determine *Aefgf* mRNA levels relative to rpS7. The combined results of four independent experiments are shown (mean +/− standard error). There were no significant differences detected between non-fed and blood-fed (*p* = 0.42), non-fed and virus-fed (*p* = 0.60), or blood-fed and virus-fed (*p* = 0.77) by paired *t*-test. (**B**) *Aefgf* and *Aefgfr* can be knocked down in Aag2 cells. dsRNAs corresponding to *Aefgf*, *Aefgfr*, or *gfp* were added to Aag2 cells and 24 h later, cells were harvested and total RNA was used for qRT-PCR. Values were normalized using the rpS7 gene and analyzed by unpaired *t*-test. (**C**) Silencing *Aefgf* or *Aefgfr* does not affect SINV replication in Aag2 cells. Either no dsRNA or dsRNAs corresponding to *Aefgf*, *Aefgfr*, or *gfp* were added to Aag2 cells and 24 h later cells were infected with MRE. Virus titers were determined at the times indicated. The data shown are the results of two replicates (+/− standard error) and significance was analyzed by repeated measures one-way ANOVA, which determined no significant differences between treatments (*p* = 0.313). (**D**) Knocking down *Aefgf* or *Aefgfr* has a negative effect on SINV titers following oral infection. Two-day old female *Ae. aegypti* were injected with either *gfp*, *Aefgf,* or *Aefgfr* dsRNA and 48 h later given a blood meal containing MRE. Mosquitoes were harvested and individually titered at 3, 5, and 7 days post blood meal. A total of between 66 and 83 mosquitoes were sampled for each treatment and time point. Significance was determined by Mann-Whitney test.

In order to study the role of FGF signaling during viral infection in mosquitoes, we first tested whether levels of *Aefgf* mRNA were altered in mosquitoes that were given a blood meal containing or lacking SINV. *Aefgf* expression was determined relative to that of mRNA for ribosomal protein S7 (rpS7) (which did not change significantly due to either treatment). No significant change in *fgf* mRNA levels was observed at any of the time points, although there was more variability in *fgf* mRNA levels in samples from blood fed mosquitoes collected at 5 days pi (dpi; Figure 1A). Thus, *Aefgf* mRNA levels do not appear to be altered by blood feeding or by oral infection with SINV.

We next sought to determine the effect of reducing FGF signaling on oral infection by SINV. To do this, we transiently silenced *Aefgf* and *Aefgfr*. Since *fgf* and *fgfr* are both single copy genes in *Ae. aegypti*, and the products from these genes work together in the same signaling pathway, we reasoned that silencing either gene should lead to a similar phenotype. Initially, we silenced *fgf* and *fgfr* in *Ae. aegypti* Aag2 cells by adding synthesized dsRNA corresponding to *Aefgf*, *Aefgfr*, or a control dsRNA (*gfp*) to the cells. After 24 h, the levels of the *Aefgf* and *Aefgfr* transcripts were reduced by 35-fold and 7-fold compared to *gfp* dsRNA, respectively, indicating that these transcripts can be knocked down successfully in *Ae. aegypti* cells (Figure 1B). Silencing of *Aefgf* or *Aefgfr* in Aag2 cells had no significant effect on 5’dsMRE16ic titers (Figure 1C), indicating that expression of the encoded proteins AeFGF and AeFGFR was not required for SINV replication at the cellular level.

However, when *Aefgf* or *Aefgfr* were silenced by injection of dsRNA in mosquitoes, titers of orally acquired 5’dsMRE16ic were significantly reduced at both 3 and 5 dpi compared to mosquitoes injected with control *gfp* dsRNA (Figure 1D). We were not able to formally demonstrate knockdown of *Aefgf* or *Aefgfr* by measuring transcript levels in whole mosquitoes, due to a high degree of variability between injected mosquitoes. However, the effects of silencing either *Aefgf* or *Aefgfr* were very similar, as would be predicted since the encoded proteins act in the same pathway. This lends confidence to the effect on virus titer being due to the reduction of expression of these genes by the dsRNA treatment. Silencing *Aefgf* or *Aefgfr* did not have a significant effect on SINV titers at 7 dpi, perhaps due to either renewed AeFGF and AeFGFR expression (transient gene silencing) or the virus overcoming its dependence on FGF signaling by this time. No effects on mosquito viability were observed following injection of any of the dsRNAs.

### 3.2. Stimulation of Mosquito Cell Motility by vFGF

**Figure 2:**
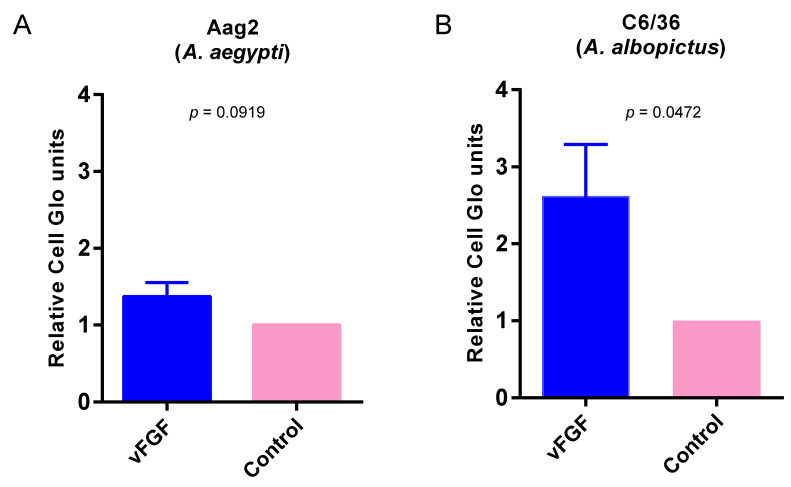
Stimulation of mosquito cell motility by viral fibroblast growth factor (vFGF). vFGF was partially purified from the supernatant of pHSFGFHA-transfected Sf9 cells and used to test its ability to stimulate transmigration of mosquito cells; control proteins were purified by the same method from supernatants of pBluescript-transfected cells. Aag2 (**A**) or C6/36 (**B**) cells were plated in the upper chamber of a transwell and cells that migrated towards vFGF or control proteins in the lower compartment of the transwell were measured by CellTiter-Glo. The bars above each column indicate the means ± standard error from three independent experiments. Significance was determined by *t* test.

Since silencing *Aefgf* or *Aefgfr* reduced SINV replication in mosquitoes, we also tested the effect of overexpressing FGF on virus replication using the recombinant SINV transducing system. Since the amount of additional sequence that can be inserted into the SINV genome without compromising virus replication is limited to about 1 kb [37] and cellular FGFs are relatively large proteins (the AeFGF ORF is more than 2.5 kbp in length), we decided to use baculovirus vFGF, whose ORF is only 0.5 kb in length. First, we verified that vFGF was able to stimulate mosquito cell motility. The ability of vFGF to induce lepidopteran insect cell motility has been previously reported [4]. However, it was not known whether vFGF would also stimulate motility of mosquito cells. To test this, vFGF was partially purified from pHSFGFHA-transfected, lepidopteran Sf9 cell supernatant using heparin-Sepharose, and its ability to stimulate cell motility was assessed as previously described [4]. Control (empty vector) transfected Sf9 cell supernatants were used as a negative control for non-specific motility. The results showed that vFGF was able to consistently stimulate migration of Aag2 and C6/36 mosquito cells (Figure 2). Although the effects were not pronounced, they are consistent with previous vFGF cell motility effects of cultured lepidopteran cells [4]. The effect on Aag2 cells was just above the standard threshold for statistical significance, but the trend between the two cell lines was consistent. Since vFGF was able to stimulate mosquito cell motility, we concluded that vFGF was able to bind and activate mosquito FGFR. 

### 3.3. Generation of Recombinant SINV Expressing vFGF 

**Figure 3:**
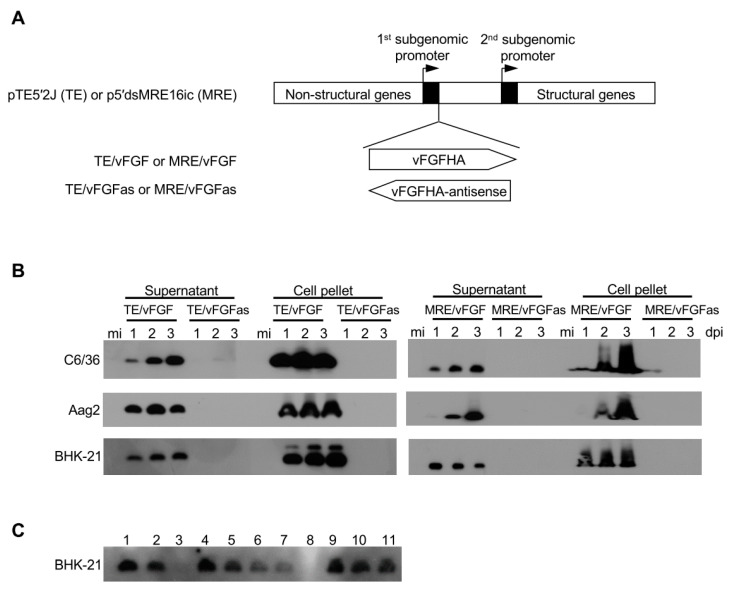
Construction of recombinant SINV and expression of vFGF. (**A**) Schematic of recombinant SINV constructions. vFGF with a C-terminal HA epitope tag was inserted into the infectious clones TE5′2J or 5′dsMRE16ic in the sense or antisense orientation under the 5′ subgenomic promoter to construct the recombinant SINV TE/vFGF, TE/vFGFas, MRE/vFGF and MRE/vFGFas. (**B**) Detection of HA-tagged vFGF expression by immunoblotting. Aag2, C6/36 and BHK-21 cells were mock-infected (mi) or infected with recombinant SINV and cells and their supernatants were harvested at 1, 2, or 3 days post-infection (dpi) to obtain lysates. vFGF was detected with anti-HA antibody. (**C**) Detection of vFGF in individual viral plaques. Plaque isolates of TE/vFGF stock virus were tested for vFGF expression by immunoblotting. Eleven plaques were picked and virus amplified in BHK-21 cells. Supernatants were collected and processed for immunoblotting.

To test whether vFGF could enhance SINV dissemination from the mosquito midgut or viral replication efficiency, recombinant viruses were generated by inserting vFGF with a C-terminal HA epitope tag into p5′dsMRE16ic (MRE) and pTE5′2J (TE), resulting in the viruses MRE/vFGF and TE/vFGF. The C-terminal tag has previously been shown to have no discernable effect on vFGF function [4,7]. As a control for insert size, the same gene was inserted in antisense orientation, which would not result in expression of vFGF (MRE/vFGFas and TE/vFGFas) (Figure 3A). Both MRE- and TE-based viruses were constructed in order to compare viruses that are either efficient (MRE) or inefficient (TE) in their ability to infect the midgut and cause disseminated infection.

To verify vFGF expression, Aag2, BHK-21, and C6/36 cells were infected with the recombinant SINVs, and supernatant and cells were analyzed by immunoblotting (Figure 3B). The expression of vFGF was detected in all cell types infected with MRE/vFGF or TE/vFGF (Figure 3B). Since vFGF is a secreted protein, it was also detected in the supernatant of infected cells (Figure 3B). 

Although the SINV transducing system has been extensively used to express heterologous proteins [24,31,38], the proportion of virus genomes in a population that retain the heterologous insert after passage has usually not been determined. To test what proportion of viruses actually expressed vFGF, immunoblotting was performed on lysates obtained from cells infected with individual plaque-derived viruses from the P2 stock of TE/vFGF used for infection experiments. The results showed that 10 out of 11 viral plaques expressed vFGF (Figure 3C; plaque 3 had weak but detectable expression), which suggested *vfgf* remained intact and was expressed by the majority of viruses in the P2 stock. 

### 3.4. Viral Growth Curve Analyses

**Figure 4:**
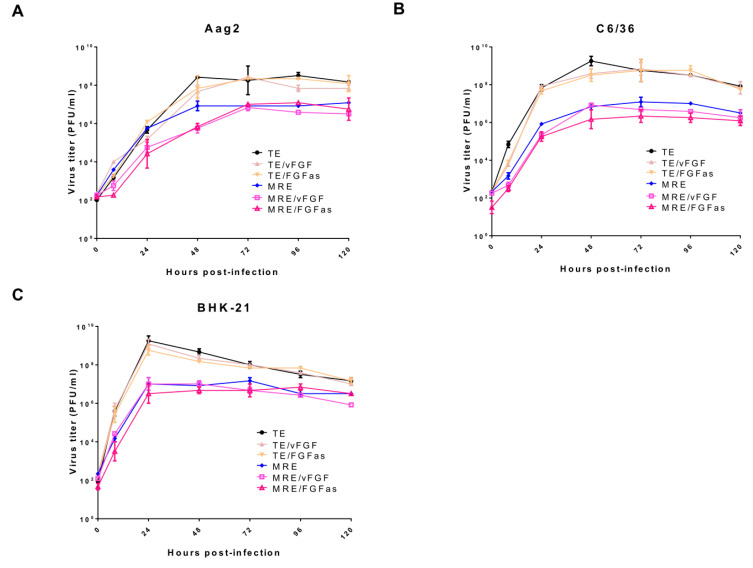
Effect of vFGF expression on SINV replication in cultured cells. Aag2 (**A**), C6/36 (**B**) and BHK-21 (**C**) cells were infected with the indicated viruses at an MOI of 0.001 PFU/cell. Supernatants from infected cells were collected at the times shown to determine virus production by TCID_50_ assays. The bars indicate the means ± standard error of three independent experiments. Results were analyzed by two-way ANOVA for Aag2 (TE viruses, *p* = 0.73; MRE viruses, *p* = 0.037), C6/36 (TE viruses, *p* = 0.56; MRE viruses, *p* = 0.13), and BHK-21 (TE viruses, *p* = 0.84; MRE viruses, *p* = 0.54).

To assess whether expression of vFGF had any effects on SINV replication in cultured cells, virus growth curves were performed. Titers of all of the viruses peaked at 48-72 hpi in Aag2 and C6/36 cells and at 24 hpi in BHK-21 cells (Figure 4). High levels of virus remained in the supernatant of the Aag2 and C6/36 cells after 48 hpi. However, virus titer dropped in BHK-21 cells after 24 hpi, probably due to cell death (BHK-21 cells are rapidly killed by SINV infection, while mosquito cells typically survive and become persistently infected). No significant differences (with one exception) were observed within each virus group in the three cell lines: TE versus TE/vFGF and TE/vFGFas, or MRE versus MRE/vFGF and MRE/vFGFas (Figure 4). The only exception was a significant difference between MRE-based viruses in Aag2 cells, but this was due to a difference between MRE and the other two viruses at early time points, and not due to expression of vFGF. The results from the TE-based and MRE-based viruses were analyzed separately, since TE-based viruses showed overall higher titers than the MRE-based viruses, as previously reported [25]. Thus, vFGF overexpression had no discernible effect on SINV replication in cultured cells.

### 3.5. Effects of vFGF Expression on Caspase Activity in SINV-Infected C6/36 and Aag2 Cells

**Figure 5:**
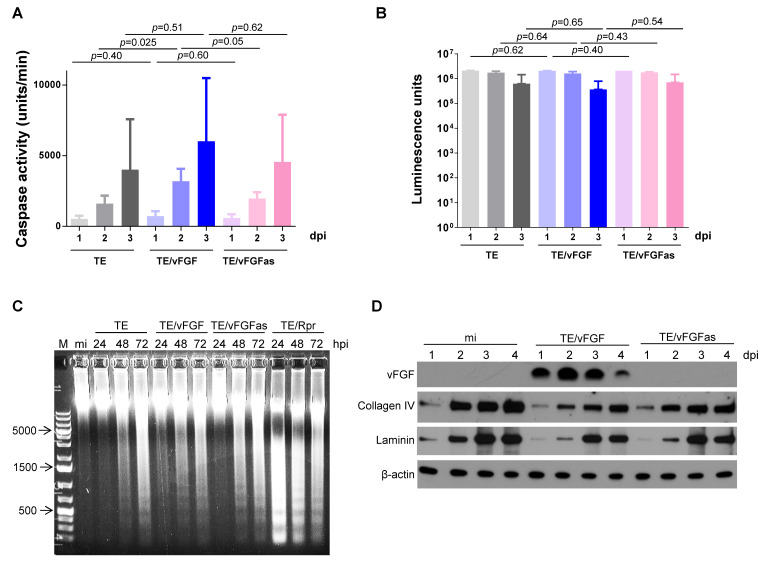
Effects of vFGF on caspase activation and cleavage of basal lamina proteins in SINV-infected cells. (**A**) Lysates from C6/36 cells infected with the indicated viruses were used to determine effector caspase activity, as measured by the cleavage rate of a fluorogenic caspase substrate (Ac-DEVD-AFC). The bars indicate the means ± standard error from four independent experiments. Significance was determined by *t* test. (**B**). Cell viability assays using the CellTiter-Glo kit were performed with C6/36 cells infected with SINV. The bars indicate the means ± standard error of three independent experiments. Significance was determined by *t* test. (**C**) C6/36 cells were mock-infected (mi) or infected with the indicated viruses and total cellular DNA was extracted and analyzed by agarose gel electrophoresis and ethidium bromide staining. The numbers to the left indicate base pairs from the marker (M) lane. (**D**). The accumulation of the proteins indicated to the left was detected by immunoblotting from supernatants of C6/36 cells infected with the indicated viruses. The blot shown is representative of three independent experiments. β-actin was detected as a loading control.

Expression of vFGF results in caspase activation in lepidopteran midguts during baculovirus infection, and caspase activity is required for basal lamina remodeling [3]. Thus, we asked whether expression of vFGF would also stimulate mosquito caspase activity during SINV infection of C6/36 cells. C6/36 cells were infected with TE, TE/vFGF, or TE/vFGFas, harvested at various time points, and cell lysates were assayed for caspase activity. No consistent significant differences in caspase activity were observed among C6/36 (Figure 5A) or Aag2 (Figure S1) cells infected by any of the three viruses. There were also no significant differences in cell viability regardless of the virus used for infections and the time of infection (Figure 5B). In all cases, there was a slight decrease in cell viability with time, consistent with a moderate cytopathic effect induced by SINV (Figure 5B). Similar results for caspase activity and cell viability were observed when C6/36 cells were infected with MRE, MRE/vFGF, or MRE/vFGFas (Figure S2). As a comparison, we compared the levels of caspase activation following infection with TE viruses versus treatment with ultraviolet light to stimulate apoptosis (Figure S3).

Although we did not observe increased caspase activation when vFGF was expressed, we also assessed whether vFGF expression resulted in increased apoptosis. As a positive control for induction of apoptosis, cytopathic effects were also examined by light microscopy and compared to C6/36 cells infected with TE/Rpr [25], a TE-based virus expressing the *Drosophila* pro-apoptotic gene reaper. C6/36 cells infected with TE, TE/vFGF, or TE/vFGFas showed cytopathic effects at late time points post-infection (pi) typical of SINV infection but did not appear to be as dramatic as the cytopathic effects caused by TE/Rpr infection, which showed extensive cell blebbing indicative of apoptosis. TE/Rpr-infected cells also exhibited genomic DNA fragmentation into oligonucleosomal ladders, characteristic of cells undergoing apoptosis (Figure 5C). The TE-, TE/vFGF-, or TE/vFGFas-infected C6/36 cells also showed DNA laddering starting at 48 hpi (Figure 5C), but it was less intense than that induced by reaper expression, and there was no obvious difference in DNA fragmentation among the three virus-infected samples indicating this phenotype was due to SINV infection and not vFGF expression. 

Since vFGF facilitates midgut escape in baculovirus-infected insects by stimulating the degradation of basal lamina proteins [3], we tested whether expression of vFGF during SINV infection of Aag2 cells had any effect on extracellular basal lamina protein accumulation. Although Aag2 cells are not of epithelial origin, we found that they do secrete laminin and collagen IV (Figure 5D). When Aag2 cells were infected with SINV and proteins in the supernatant were immunoblotted with antibodies against laminin and collagen IV, accumulation of these proteins was reduced in infected samples compared to mock-infected cells (Figure 5D). However, there was no difference in collagen IV/laminin abundance between TE/vFGF- and TE/vFGFas-infected cells (Figure 5D), again indicating that this reduction was due to SINV infection and not expression of vFGF. 

### 3.6. Effect of vFGF Expression on Mosquito Midgut Infection

**Figure 6:**
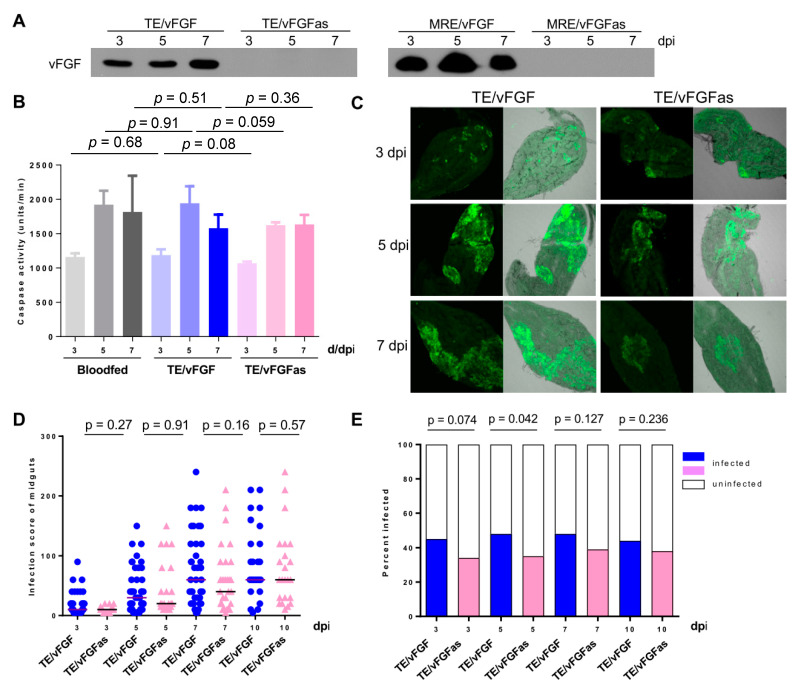
Effect of vFGF on mosquito midgut infections. (**A**) Detection of vFGF expression in SINV-infected midguts by Western blot. (**B**) Lysates from SINV-infected midguts were used to measure caspase activity by measuring the cleavage rate of a fluorogenic caspase substrate (Ac-DEVD-AFC). Significance was determined by *t*-test. (**C**) SINV infection in midguts (green) was visualized using an anti-SINV E1 antibody. Mock-infected midguts showed only weak background levels of fluorescence, similar to the uninfected regions of midgut shown. (**D**) Individual midgut infection scores were determined from anti-SINV antibody staining. Only infected midguts were included in the infection score analysis and Mann-Whitney U tests were used to determine *p* values. (**E**) Prevalence of infection in midguts was determined after anti-SINV antibody staining (positive) or uninfected (negative). Significance was determined by Fisher’s Exact test.

Since the MRE strain efficiently infects *Ae. aegypti* by the oral route, we reasoned that if vFGF expression had a positive effect on infection of mosquitoes, it would be more evident using the TE-based viruses, which do not establish midgut infection or disseminated infection as efficiently as the MRE strain. Therefore, in order to test whether expression of vFGF could affect SINV infection in mosquitoes, we orally infected mosquitoes with TE/vFGF or TE/vFGFas along with a blood meal. vFGF was expressed in mosquito midguts infected with TE/vFGF (Figure 6A) but no significant difference in midgut caspase activity was observed in the presence or absence of vFGF expression when compared to that of blood-fed, non-infected mosquitoes (Figure 6B). However, caspase activity in the midgut increased between 3 and 5 days after blood meal, regardless of the presence or absence of virus. This is consistent with previous results using chikungunya-infected mosquitoes [39].

The ability of TE/vFGF and TE/vFGFas to infect mosquito midgut cells, the primary site of infection, was compared in a semi-quantitative way by immunofluorescence using an antibody specific to E1 of SINV [40]. Each SINV-infected midgut was assigned an infection score as described in Methods. Representative examples of midgut immunofluorescence are shown (Figure 6C). No significant differences in infection score were observed between TE/vFGF and TE/vFGFas at any time point examined (Figure 6D). Prevalence of infection, as determined by the percentage of midguts that were positive for SINV antigen, was consistently higher in mosquitoes infected with TE/vFGF than TE/vFGFas but only reached statistical significance at 5 dpi (Figure 6E). 

### 3.7. Effect of vFGF Expression on SINV Dissemination in Mosquitoes

**Figure 7:**
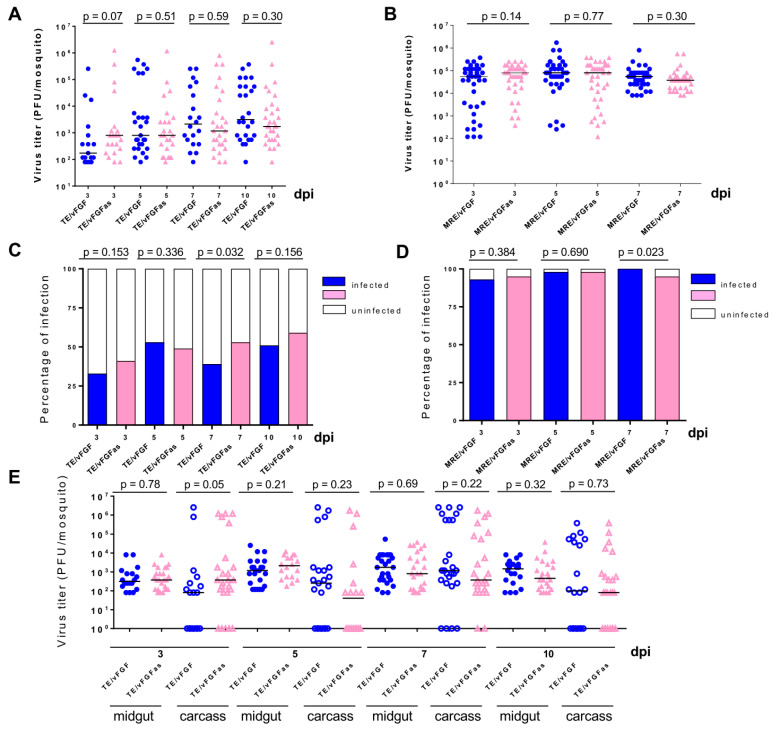
Expression of vFGF does not increase SINV replication and dissemination in mosquitoes. TE/vFGF or TE/vFGFas (**A**) and MRE/vFGF or MRE/vFGFas (**B**) replication in individual mosquitoes was measured by TCID_50_ assays. Only infected mosquitoes were included in the analyses and Mann-Whitney U tests were used to determine *p* values. The prevalence of virus infection of TE/vFGF or TE/vFGFas (**C**) and MRE/vFGF or MRE/vFGFas (**D**) in mosquitoes based on titered samples was obtained. Significance was determined by Fisher’s Exact test. (**E**) Individual midguts were titered separately from the carcass of the mosquitoes. Only carcasses with a corresponding infected midgut were included in the analysis. Mann-Whitney U tests were used to determined *p* values.

To determine if vFGF had any effects on the levels of SINV replication in mosquitoes, titers from individual TE/vFGF-infected mosquitoes were compared to those infected with the antisense control virus TE/vFGFas. We also compared infection by MRE/vFGF and MRE/vFGFas in these experiments. No effects on mosquito viability were observed following infection with any of the viruses during the time frame of the experiment. Virus titers and infection prevalence (as determined by detectable virus titer) were similar between mosquitoes infected with TE/vFGF and TE/vFGFas (Figure 7A,C) or between MRE/vFGF and MRE/vFGFas (Figure 7B,D). Although at 7 dpi there were significant prevalence differences between vFGF- and vFGFas-containing viruses, these did not have a consistent pattern. In order to determine if expression of vFGF during SINV infection had any advantage in promoting virus escape from the midgut, SINV-infected mosquitoes were dissected and individual midguts and carcasses (the rest of the mosquito excluding the midgut) of the mosquitoes were titered separately. vFGF expression did not appear to affect SINV dissemination from the midgut, as neither midgut nor carcass titers were significantly different between the two viruses at any of the time points examined, with the possible exception of 3 dpi carcass (Figure 7E). 

## 4. Discussion

Insights into how baculoviruses escape the midgut barrier of their insect hosts have come from studies of the baculovirus-encoded *vfgf* gene. These studies revealed that the vFGF protein triggers a signal transduction pathway in lepidopteran insects, involving a stepwise cascade of proteinase activation, i.e., matrix metalloproteases activate effector caspases, which then act extracellularly to degrade basal lamina protein components, allowing baculoviruses to infect susceptible tracheal cells prior to the regeneration of the tracheal basal lamina [3]. Because mosquito-borne arboviruses also face the problem of breaching the midgut basal lamina after oral infection, components of this pathway may also be utilized by arboviruses for midgut escape. We hypothesized that either blood feeding or arbovirus infection may lead to increased FGF signaling in the midgut and subsequent basal lamina remodeling, which could promote midgut escape. Indeed, recent evidence indicates that the midgut basal lamina undergoes changes following a blood meal that are consistent with a remodeling process [17,18,19]. Although we did not observe significant changes in *fgf* transcript levels after blood feeding, this does not necessarily mean that FGF signaling is not affected at a posttranscriptional level. Indeed, a known mechanism of FGF regulation consists of FGF ligand being sequestered by binding to heparin on cell surfaces and being released by protease activation when cells need to initiate FGF signaling [41].

To test whether FGF signaling was important in arbovirus infection of the mosquito midgut or escape from the midgut, we examined the effects of reducing or increasing FGF expression levels on SINV replication in vivo and in vitro. Silencing of either *Aefgf* or *Aefgfr* expression in *Ae. aegypti* mosquitoes reduced the ability of SINV to replicate following oral infection, consistent with FGF signaling being required for optimal virus replication. The fact that a similar outcome was observed when either *Aefgf* or *Aefgfr* was silenced lends confidence to the conclusion that FGF signaling is involved in optimal virus replication following oral infection. Since silencing either *Aefgf* or *Aefgfr* did not affect SINV replication in Aag2 cells, this suggests that the requirement for FGF signaling is not at the cellular level, but instead is at the level of tissue, organ, or organism. One possibility is that reducing FGF signaling may delay the ability of the virus to escape the midgut and cause disseminated infection. Whether the decreased titers observed in mosquitoes with reduced FGF or FGFR expression are due to a defect in midgut escape remains to be determined. It is worth noting that although no increase in caspase activity was detected in midguts of infected mosquitoes compared to mosquitoes that received a non-infectious blood meal, blood feeding itself does induce higher caspase activity in mosquito midguts than in midguts from non-blood-fed mosquitoes [39]. Thus, SINV may take advantage of an increase in FGF signaling that is triggered by a blood meal, which would be expected to result in increased caspase activity and basal lamina reorganization, in order to more efficiently escape the midgut and establish systemic infection. 

We also observed that overexpression of baculovirus vFGF during SINV replication in cells or mosquitoes resulted in no differences in caspase activity, virus replication, or dissemination. We cannot rule out the possibility that vFGF is unable to fully function in mosquito cells, although our cell migration data suggest that it can stimulate motility of cultured mosquito cells. Another, and in our view more likely, possibility is that endogenous FGF levels are already sufficient for basal lamina reorganization, and thus overexpression of vFGF does not increase basal lamina reorganization any further. Similarly, there are potential explanations for the observation that stimulation of Aag2 cell motility by vFGF was relatively low. For example, Aag2 cells already express the FGF transcript (Figure 1B), so exposing them to additional FGF may not have a large effect on cell motility. Another possibility is that Aag2 cells may have low sensitivity to FGF if they have low levels of heparan sulfate on their surface, since it has been shown that levels of heparan sulfate on the cell surface can determine the sensitivity of cells to FGF signals by influencing receptor binding [42]. Interestingly, SINV infection resulted in decreased accumulation of basal lamina proteins, collagen IV, and laminin, in the supernatant of Aag2 cells. Whether this is an effect on expression, secretion, or degradation of these proteins is not known. 

In summary, we observed that reducing FGF or FGFR expression had a negative effect on SINV replication in *Ae. aegypti* mosquitoes, but overexpressing vFGF did not increase SINV replication. These results are consistent with the hypothesis that the FGF pathway is involved in one or more steps in the infection of *Ae. aegypti* by SINV, such as promoting midgut escape, but additional tests of this hypothesis are needed. Manipulation of the appropriate caspases or matrix metalloproteases in the mosquito may provide more insights into the mechanisms of arbovirus midgut escape. 

## Supplementary Materials

The following are available online at https://www.mdpi.com/1999-4915/12/9/943/s1, Figure S1: Caspase activity in Aag2 cells infected with TE or TE/vFGF. Figure S2: aspase activity and cell viability of C6/36 cells infected with MRE, MRE/vFGF, or MRE/vFGFas. Figure S3: aspase activity in Aag2 cells infected with TE viruses or treated with UV light as a positive control for caspase activation.
