# Supplementary material for: Effects of Manipulating Fibroblast Growth Factor Expression on Sindbis Virus Replication In Vitro and in Aedes aegypti Mosquitoes
Source: Viruses. 2020 Aug 26;12(9):943. doi: 10.3390/v12090943 (PMC7552049; doi:10.3390/v12090943)
Supplement: Supplementary file 1 [file viruses-12-00943-s001.pdf]

Supplemental Figures

Wu et al., Effects of manipulating fibroblast growth factor expression on Sindbis virus replication *in vitro* and in *Aedes aegypti* mosquitoes

Fig. S1. Caspase activity in Aag2 cells infected with TE or TE/vFGF. Cells were infected with TE or TE/vFGF and caspase activity was analyzed at the times shown (hours post infection or hpi) using ac-DEVD-AFC as described in the Methods. Significance was determined by t test.

Fig. S2. Caspase activity and cell viability of C6/36 cells infected with MRE, MRE/vFGF, or MRE/vFGFas. Cells were infected with the indicated viruses and caspase activity or cell viability was determined as described in the Methods at the times shown (days post-infection, or dpi). Significance was determined by t test.

Fig. S3. Caspase activity in Aag2 cells infected with TE viruses or treated with UV light as a positive control for caspase activation. The data shown for the virus-infected cells are the same data that are presented in Fig. S1.

Fig. S1

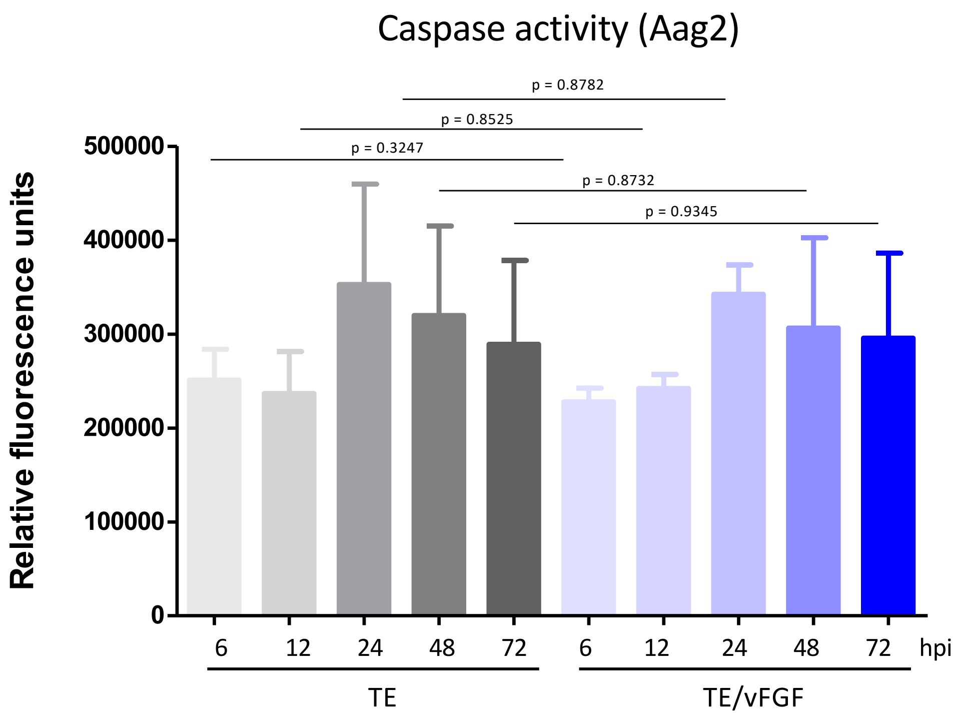

Caspase activity (Aag2)
p = 0.8782
p = 0.8525
p = 0.3247
p = 0.8732
p = 0.9345
Relative fluorescence units
500000
400000
300000
200000
100000
0
6
12
24
48
72
6
12
24
48
72
hpi
TE
TE/vFGF

Fig. S2

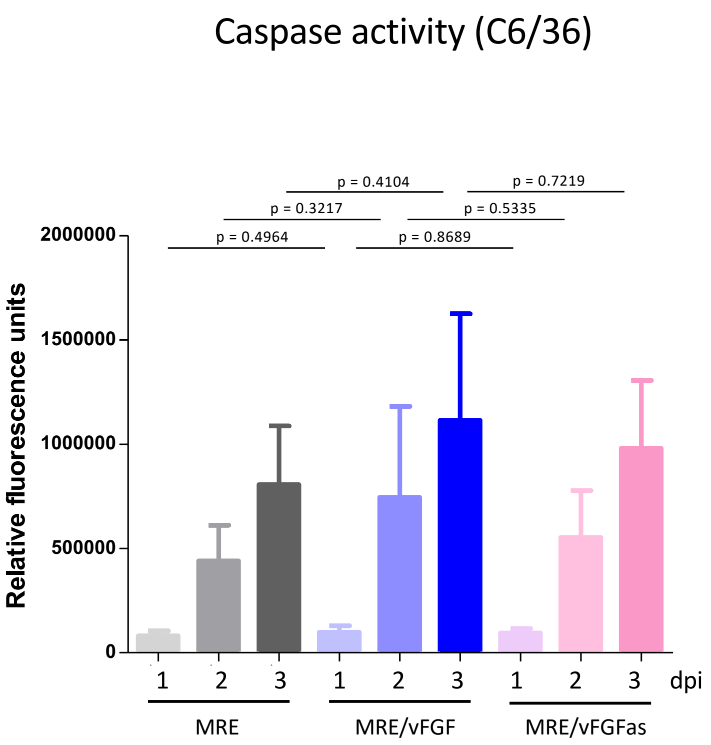
Caspase activity (C6/36)
p = 0.4104
p = 0.7219
p = 0.3217
p = 0.5335
p = 0.4964
p = 0.8689
Relative fluorescence units
2000000
1500000
1000000
500000
0
1 2 3 1 2 3 1 2 3 dpi
MRE
MRE/vFGF
MRE/vFGFas

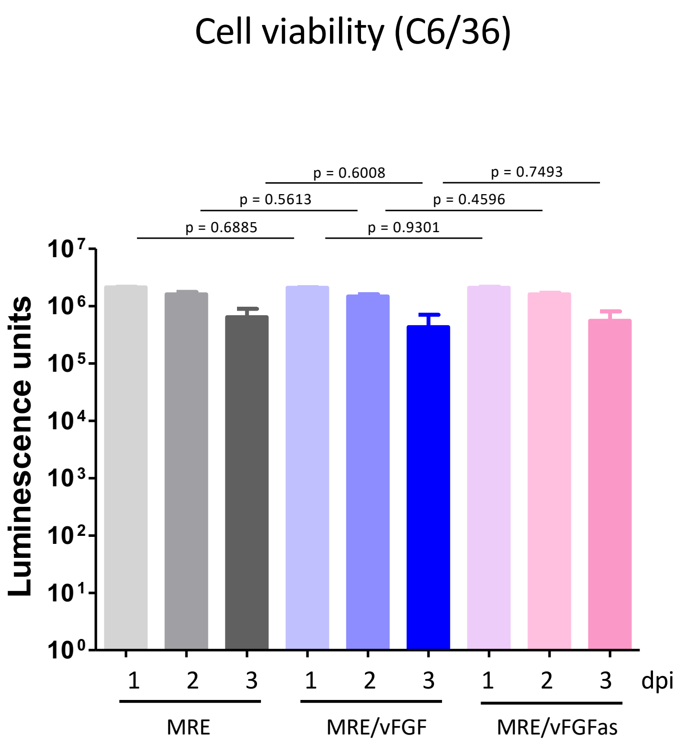
Cell viability (C6/36)
p = 0.6008
p = 0.7493
p = 0.5613
p = 0.4596
p = 0.6885
p = 0.9301
Luminescence units
10^7
10^6
10^5
10^4
10^3
10^2
10^1
10^0
1 2 3 1 2 3 1 2 3 dpi
MRE
MRE/vFGF
MRE/vFGFas

Fig. S3

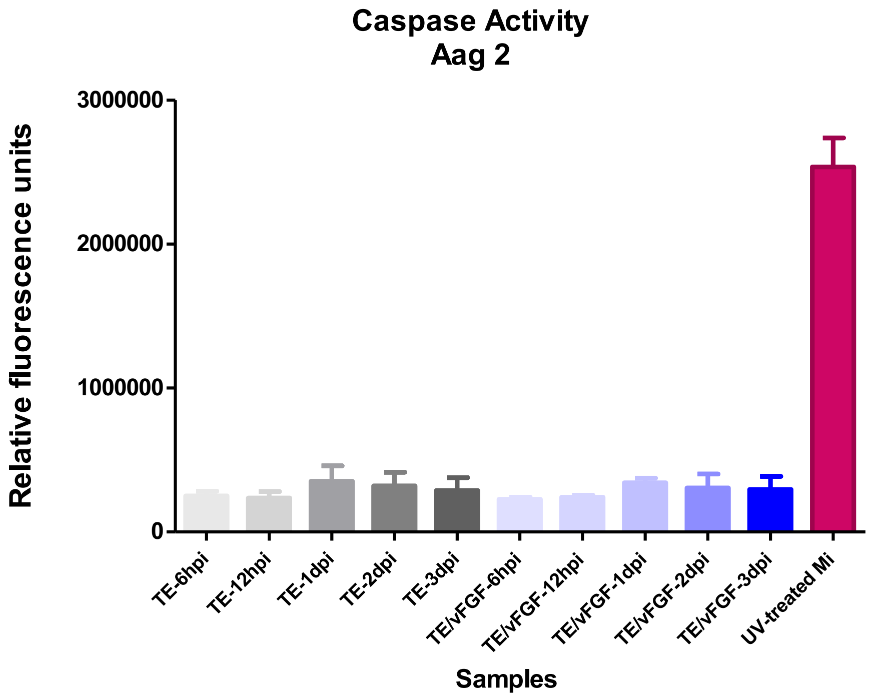
Caspase Activity
Aag 2
Relative fluorescence units
3000000
2000000
1000000
0
TE-6hpi
TE-12hpi
TE-1dpi
TE-2dpi
TE-3dpi
TE/vFGF-6hpi
TE/vFGF-12hpi
TE/vFGF-1dpi
TE/vFGF-2dpi
TE/vFGF-3dpi
UV-treated Mi
Samples
